# Biodistribution of co-exposure to multi-walled carbon nanotubes and nanodiamonds in mice

**DOI:** 10.1186/1556-276X-7-473

**Published:** 2012-08-23

**Authors:** Qi Wei, Li Zhan, Bi Juanjuan, Wang Jing, Wang Jianjun, Sun Taoli, Guo Yi’an, Wu Wangsuo

**Affiliations:** 1 Radiochemical Laboratory, Lanzhou University, Lanzhou, Gansu, 730000, China; 2 Institute of Modern Physics, Chinese Academy of Sciences, Lanzhou, Gansu, 730000, China; 3 School of Pharmacy, Lanzhou University, Lanzhou, Gansu, 730000, China; 4 State Key Laboratory of Applied Organic Chemistry, Lanzhou University, Lanzhou, Gansu, 730000, China

**Keywords:** oMWCNTs, NDs, Biodistribution, Excretion

## Abstract

In this work, technetium-99 (^99m^Tc) was used as the radiolabeling isotope to study the biodistribution of oxidized multi-walled carbon nanotubes (oMWCNTs) and/or nanodiamonds (NDs) in mice after intravenous administration. The histological impact of non-radiolabeled oMWCNTs or NDs was investigated in comparison to the co-exposure groups. ^99m^Tc-labeled nanomaterials had high stability *in vivo* and fast clearance from blood*.* After a single injection of oMWCNTs, the highest distribution was found in the lungs, with lower uptake in the liver/spleen. As for NDs injected alone, high distribution in the liver, spleen, and lungs was observed right after. However, uptake in the lungs was decreased obviously after 24 h, while high accumulation in the liver or spleen continued. After co-injection of oMWCNTs and NDs, oMWCNTs significantly affected the distribution pattern of NDs *in vivo*. Meanwhile, the increasing dose of oMWCNTs decreased the hepatic and splenic accumulation of NDs and gradually increased lung retention. On the contrary, the NDs had no significant effects on the distribution of oMWCNTs in mice. Histological photographs showed that oMWCNTs were mainly captured by lung macrophages, and NDs were located in the bronchi and alveoli after co-administration. oMWCNTs and NDs had different modes of micro-cells. In conclusion, the behavior and fate of NDs in mice depended strongly on oMWCNTs, but NDs had a small influence on the biodistribution and excretion pattern of oMWCNTs.

## Background

The fields of nanoscience and nanotechnology have developed rapidly with sustainable interest in submicron materials, about their assembly, and unique properties. A wide range of carbon nanomaterials, such as fullerenes, nanotubes, graphene, and nanodiamonds (NDs) has been studied for potential applications in the diagnosis and treatment of diseases [1-4]. Therefore, toxicity and biosafety of carbon nanomaterials must be investigated deeply for safe application in biomedicine in the future. The studies of biodistribution are the precondition for further understanding of the mechanism of biological toxicity, behavior, and fate of nanomaterials in animals *in vivo*.

It is reported that the biodistribution of carbon nanomaterials depends strongly on various factors such as nanoparticle size, type of functionalization, concentration, duration of exposure, administration method, and even dispersant [5-11]. However, it is still unclear whether the nanostructure is certain to affect and determine the behavior and fate of carbon nanomaterials *in vivo*. Meanwhile, another further issue of interest is clinical application of nanodrugs in the future. Thus, it is important to further investigate the biodistribution of nanomaterials post co-exposure to different structures of carbon nanomaterials. Recently, Li et al. [12] studied the biodistribution of oxidized multi-walled carbon nanotubes (oMWCNTs) and oxidized graphene with co-injection in mice. The results showed that carbon nanotubes could affect strongly the biodistribution of oxidized graphene, but oxidized graphene could not affect the biodistribution of carbon nanotubes. However, the detailed mechanism has not been discussed theoretically. In order to confirm the experimental results and further understand the experimental mechanism, oMWCNTs and NDs are selected, and their biodistribution after co-injection *in vivo* are to be studied in this work.

Radioisotope tracing technology can measure accurately the content of carbon nanomaterials in any tissue. It has been widely used to collect reliable experimental data in the researches of biodistribution, biotoxicity, and related metabolism [13]. Herein, oMWCNTs and NDs were radiolabeled with technetium-99 (^99m^Tc) (*T*_1/2_ = 6.02 h, *E*_g_ = 141 keV). On the basis of stability studies, the labeled compounds were injected into mice individually or combinedly so as to study their tissue distribution and interaction *in vivo*. Moreover, the results would be compared with previous reports to confirm whether one carbon nanomaterial can really affect the behavior and fate of another *in vivo* and to further understand the related mechanism.

## Methods

### Preparation of oxidized MWCNTs and NDs

Multi-walled carbon nanotubes (MWCNTs) prepared by chemical vaporization deposition were commercially obtained from Shenzhen Nanotech Port Co. Ltd., Guangdong, China. According to the product specification, as-received MWCNTs were determined with transmission electron microscopy (TEM) to be 1 to 10 nm in length, with a diameter of 10 to 30 nm. Purity was >96% (wt.%), containing <3% amorphous carbon and <0.2% ash. The as-grown MWCNTs (named as untreated MWCNTs) were added into the solution of 3 mol/L HNO_3_ to remove the hemispherical caps of the nanotubes. The mixture of 3 g MWCNTs and 400-mL 3 mol/L HNO_3_ was ultrasonically stirred for 24 h. The suspension was filtrated and rinsed with deionized water until the pH of the suspension reached about 6, and it was then dried at 80°C. Thus, the treated MWCNTs (named oMWCNTs) were calcined at 450°C for 24 h to remove the amorphous carbon [14].

NDs with individual sizes ranging from 2 to 10 nm, generously supplied by Gansu Gold Stone Nano. Material Co., Ltd. (Lanzhou, China), were synthesized by deto-nation techniques. As-received NDs were characterized by a series of spectroscopy techniques and analytical methods. Fourier transform-infrared spectroscopy (FT-IR, Nicolet Avatar-360, America Nicolet.) showed that the NDs were functionalized with -OH and -COOH on the surface. The NDs were used directly without any treatment. Particle size analysis was performed for the supernatant and precipitate of NDs after centrifuging for 10 min in 3,000 rpm.

### ^99m^Tc labeling of oMWCNTs/NDs and determination of labeling yields

The ^99m^Tc labeling of oMWCNTs/NDs and determination of labeling yields were preformed (^99m^TcO_4_^−^ was purchased from the China Institute of Atomic Energy, Beijing, China; 5 mCi) according to the method of Li et al. [5]. The unfilled electron orbits of Tc (V) were filled immediately by electrons donated by four hydroxy groups from oMWCNTs/NDs, and thus, a stable chelate complex ^99m^Tc-oMWCNTs/NDs was formed. In brief, oMWCNTs or NDs were dissolved in deionized water with ultrasonic device for 5 min, and then ascorbic acid, stannous chloride, and ^99m^TcO_4_^−^ were added into the suspension. This mixture was stirred at 90°C for 20 min. After centrifugation, the supernatant was decanted, and the remaining solid was ^99m^Tc-oMWCNTs or NDs. The radiolabeling yields of oMWCNTs and NDs were showed in Additional file 1: Figure S1. Due to the short lifetime of ^99m^Tc (6.02 h), the authors had to measure the radio counts in 1 day for accurate determination of the tag; otherwise, the tag (^99m^Tc) would decay out in 10 half times.

### Stability of ^99m^Tc-oMWCNTs/NDs

To determine the stability of ^99m^Tc-oMWCNTs/NDs, the radiolabeling yields of ^99m^Tc-oMWCNTs/NDs were measured at 0.5, 1.5, 15, and 24 h after synthesis by paper chromatography using Whatman 1 paper strips (Maidstone, Kent, UK). A sample of the test solution (10 μL) was applied at 1 cm from the lower end of the strips. The strips were developed in normal saline until the solvent reached the top portion. The strips were dried and cut into 1-cm segments, and the distribution of radioactivity on the strip was determined using a gamma-ray counter.

### Biodistribution study in mice

Kunming mice (female) initially weighing 15 to 18 g were provided by Laboratory Center for Medical Science, Lanzhou University, Gansu, China. All animals were housed in individual cages in a temperature (21°C to 22°C) and light (on from 08:00 to 20:00 hours) controlled environment and were fed food and tap water *ad libitum*. Mice were cared for and used humanely according to the Animal Care and Use Program guidelines of Lanzhou University.

In this study, each mouse in all 16 groups (five mice per group) was intravenously injected by the tail vein with 0.2 mL (500 μg) of ^99m^Tc-oMWCNTs, ^99m^Tc-NDs, ^99m^Tc-oMWCNTs + NDs, and ^99m^Tc-NDs + oMWCNTs suspension (pH = 7.25, *C*_NaCl_ = 0.9%), respectively. They were sacrificed at 2, 8, 16, and 24 h post injection, and their tissues of organs, such as the heart, lung, liver, spleen, kidney, and stomach, were immediately dissected, and blood and mixture of feces and urine were collected. Each tissue was wrapped in foil, weighted, and counted for ^99m^Tc. Data were corrected for physical decay of radioactivity. Distribution in tissue was presented in percent injected dose per gram of wet tissue (%ID/g), which could be calculated by the percent injected dose (tissue activity/total activity dose) per gram of wet tissue [6]. The excretion and blood clearance rate of nanoparticles from mice was investigated by ^99m^Tc in urine, feces, and blood of mice at different time intervals from 0 to 25 h post dosing. Results were expressed as percent injected dose per gram of wet tissue. To study the effects of the nanoparticles’ dose, each mouse in all 24 groups (five mice per group) was intravenously injected by the tail vein with 0.2 mL of ^99m^Tc-NDs (500 μg), ^99m^Tc-oMWCNTs (100 μg) + NDs (500 μg), ^99m^Tc-oMWCNTs (500 μg) + NDs (500 μg), ^99m^Tc-oMWCNTs (500 μg), ^99m^Tc-NDs (100 μg) + oMWCNTs (500 μg), and ^99m^Tc-NDs (500 μg) + oMWCNTs (500 μg). They were sacrificed at 2, 8, 16, and 24 h post injection and treated with the same method as above.

### Histopathological morphology studies

Three groups (three mice per group) were sacrificed at 2 h post intravenously (i.v.) with 800 μg oMWCNTs, NDs, oMWCNTs + NDs (pH = 7.28, *C*_NaCl_ = 0.9%, as control). Some tissues of organs, including the liver, lungs, kidneys, and spleen, were immediately collected. These tissues were fixed in 10% buffered formalin and processed for routine histology with hematoxylin and eosin stain by the Center for Medical Science, Lanzhou University (Lanzhou, China). Microscopic observation of tissues was carried out with an Olympus Microphot-CX41 microscope (Olympus Corporation, Shinjuku, Tokyo, Japan) coupled with a digital camera.

The tissues with high radioactivity retention, of the lungs, liver, and spleen, were selected and digested by a mixture of HClO_4_ and H_2_O_2_ to perform the TEMs.

## Results and discussion

### Results

#### Preparations of oMWCNTs and NDs

**Figure 1:**
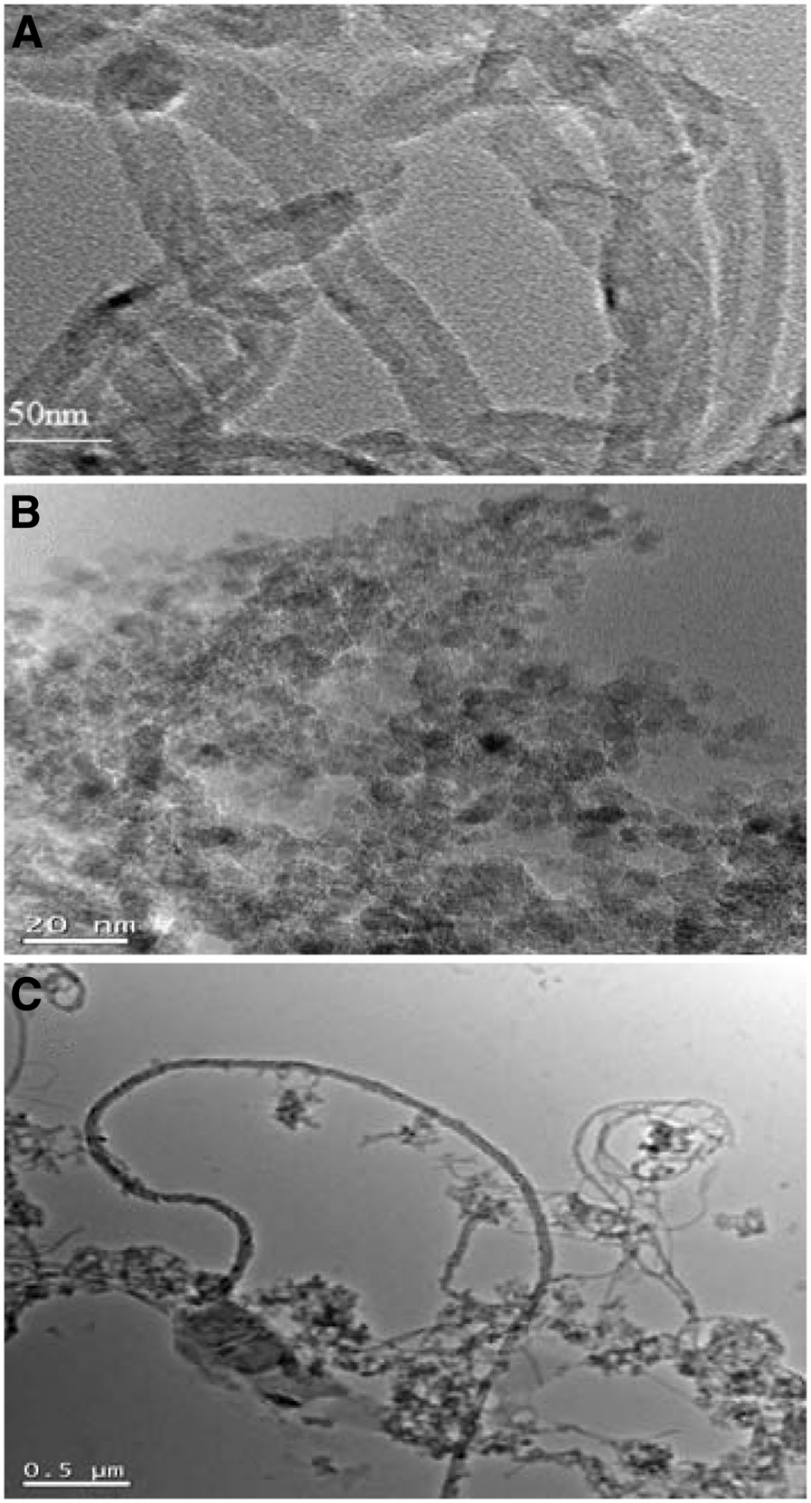
**TEM images of oMWCNTs (A), NDs (B), and oMWCNTs + NDs (C).** oMWCNTs and NDs were suspended in water before the TEM grids were prepared.

The TEM of oMWCNTs, NDs, and oMWCNTs + NDs were shown in Figure 1. It indicated that the hollow tubular structures of oMWCNTs were observed clearly with diameters of 20 to 30 nm. As observed in Figure 1B and Additional file 1: Figure S7, approximately 16.16% of NDs used in this experiment were less than 65 nm, and 83.84% of the rest was agglomerated in water with a larger size of approximately 217 nm. Figure 1C showed that NDs have been connected to the surface of oMWCNTs in mixed solution. The FT-IRs (Additional file 1: Figure S6) indicated that oMWCNTs and NDs contained a large amount of -COOH and -OH.

#### Stability of ^99m^Tc-oMWCNTs/NDs in vitro and in vivo

Additional file 1: Figure S2 showed that the labeling yield of ^99m^Tc-oMWCNTs/NDs was more than 90% and was almost invariant within 24 h *in vitro.* Therefore, the radioactive counts of ^99m^Tc would be able to reflect the behavior and fate of carbon nanoparticles *in vivo* in mice.

#### Biodistribution study in mice

**Figure 2:**
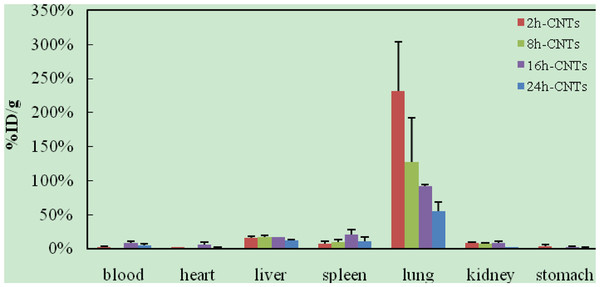
**Biodistribution of oMWCNTs in mice at 2, 8, 16, and 24 h post i.v. with **^**99m**^**Tc-oMWCNTs.**

**Figure 3:**
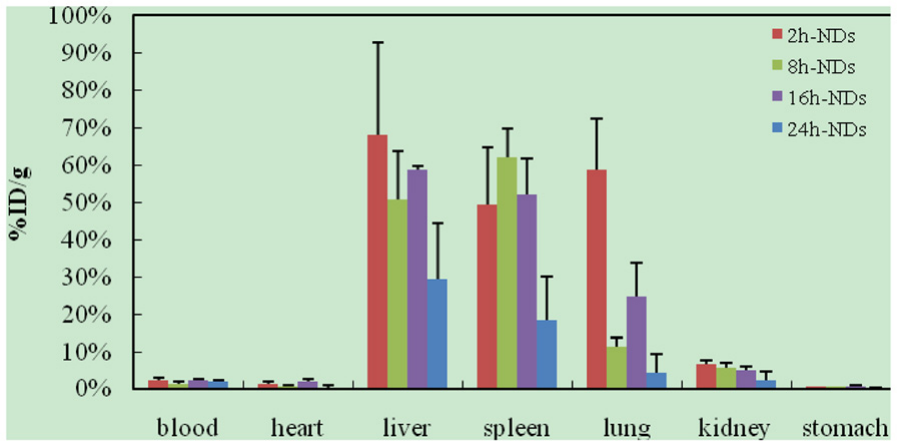
**Biodistribution of NDs in mice at 2, 8, 16, and 24 h post i.v. with **^**99m**^**Tc-NDs.**

**Figure 4:**
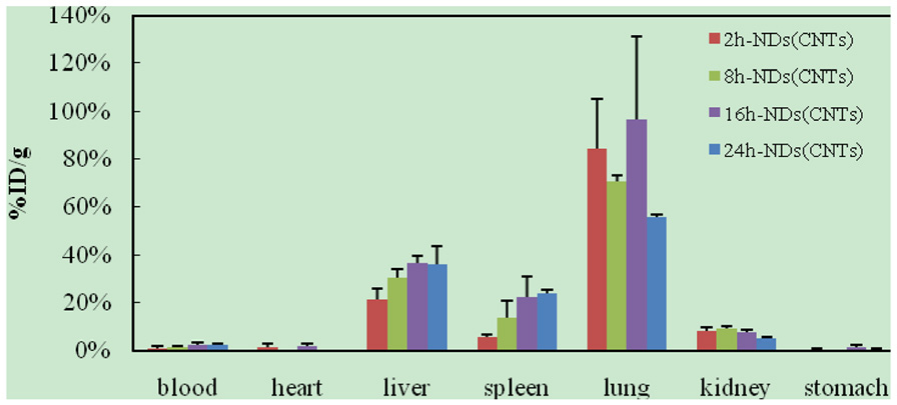
**Biodistribution of NDs in mice at 2, 8, 16, and 24 h post i.v. with **^**99m**^**Tc-NDs + oMWCNTs.**

**Figure 5:**
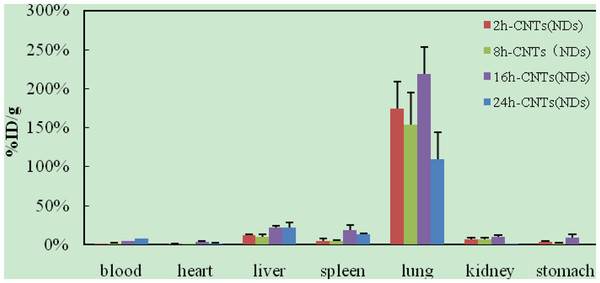
**Biodistribution of oMWCNTs in mice at 2, 8, 16, and 24 h post i.v. with **^**99m**^**Tc-oMWCNTs + NDs.**

After intravenous injection of ^99m^Tc-oMWCNTs/NDs, the biodistribution results could be observed in Figures 2 and 3. The results (Figure 2) indicated that most oMWCNTs were accumulated in the lungs, with just a small amount of retention in the liver and spleen, and that oMWCNTs could be eliminated slowly from the tissues with time process. The highest uptake could be observed in the liver, lungs, and spleen within 24 h after a single administration of ^99m^Tc-NDs (Figure 3). Interestingly, high uptake values of approximately 58.7% after 2 h dropped to approximately 10%ID/g after 8 h in the lungs, but uptake of the spleen increased clearly. Part of the NDs could be eliminated within 24 h after single injection. Uptake of the liver and spleen at 24 h post i.v. were approximately 30% and 20%, respectively, but less than 5% in other tissues. As can be seen in Figure 4, the biodistribution of NDs post co-exposure to the mixture of ^99m^Tc-NDs and oMWCNTs in 24 h was similar to that in Figure 2 but different with that Figure 3; therefore, this result indicated clearly that oMWCNTs would obviously change the biodistribution pattern of NDs *in vivo*. In co-exposure, we could observe the highest distribution of NDs in the lungs and lower distribution, in the liver and spleen, after 2 h. However, values for the spleen and liver increased slowly with time at 8, 16, 24 h after administration. Kidney uptake was less than 5% ID/g during the observation time. On the other hand, the biodistribution pattern of oMWCNTs (Figure 5) in co-injection was similar to that in single injection (Figure 2), so NDs did not affect the behavior and fate of oMWCNTs *in vivo*.

**Figure 6:**
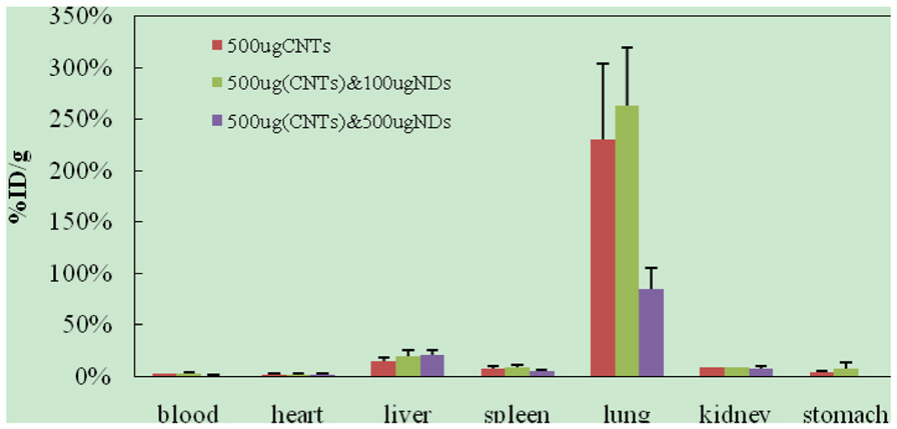
**Effect of NDs doses on biodistribution of 500 μg **^**99m**^**Tc-oMWCNTs in mice 2 h after co-injection.**

**Figure 7:**
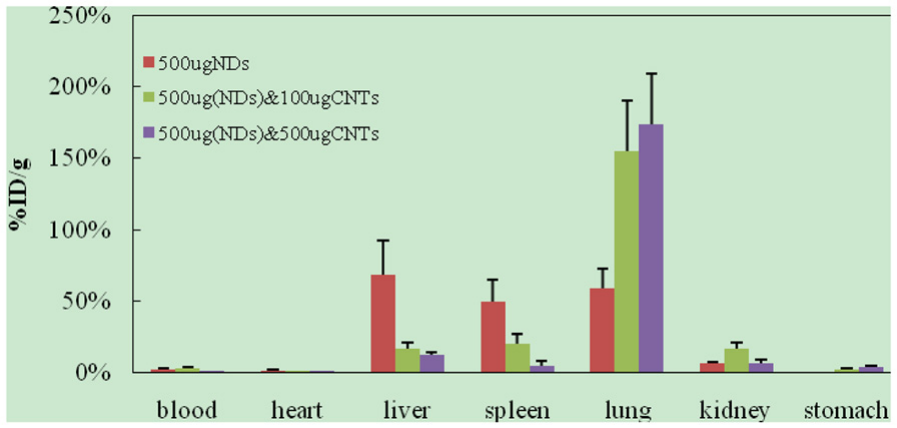
**Effect of oMWCNTs doses on biodistribution of 500 μg **^**99m**^**Tc-NDs in mice 2 h after co-injection.**

From Figure 6, we found that the doses of NDs could obviously affect tissue uptakes of oMWCNTs after post exposure to a mixture of 500 μg of oMWCNTs and different doses of NDs in 2 h. Meanwhile, with increasing of the dose of oMWCNTs from 0 to 500 μg, the oMWCNTs could improve the tissue uptakes of NDs (Figure 7).

#### Histology of mice tissues

**Figure 8:**
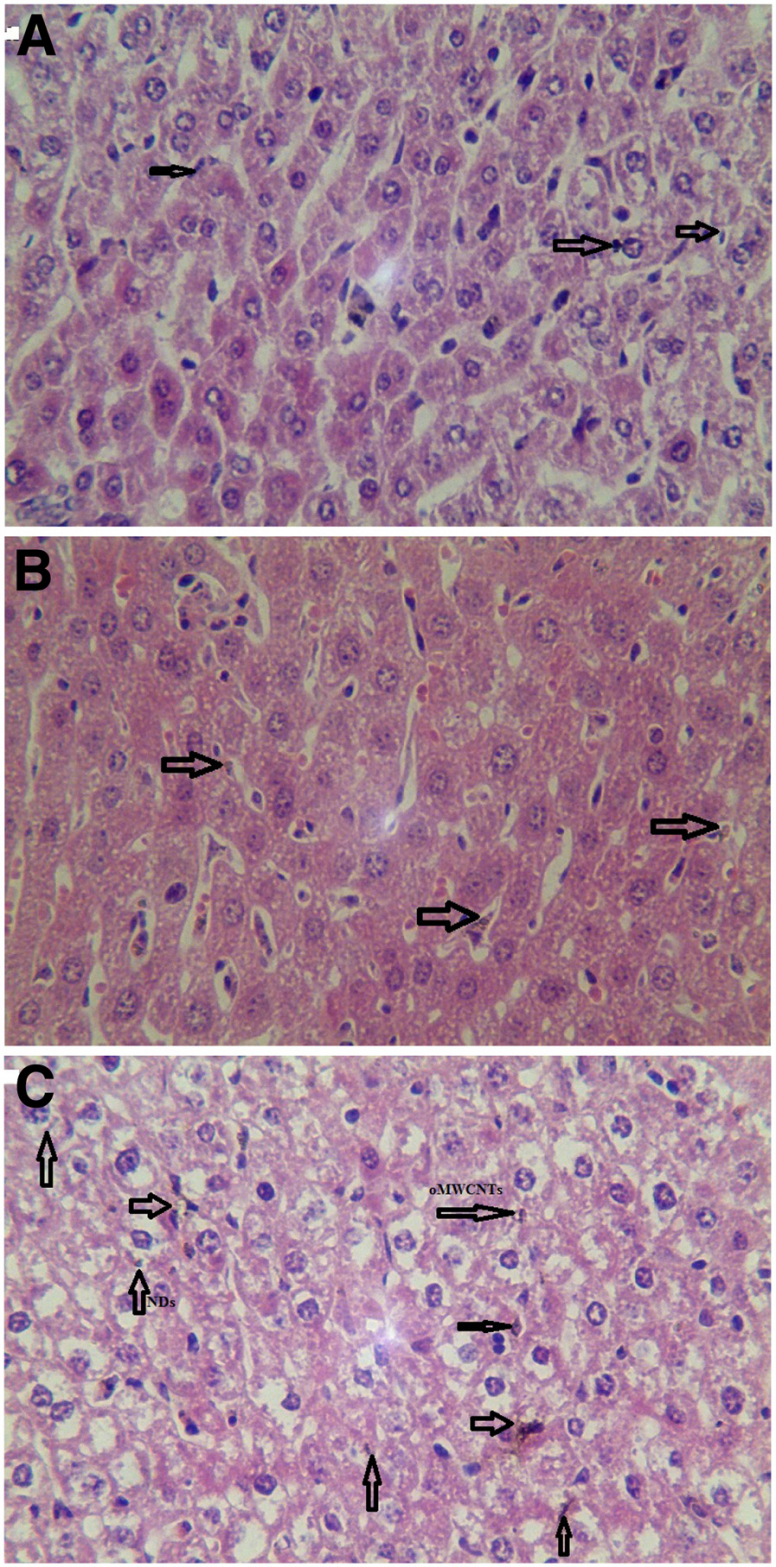
**The histology of liver tissues (×20).** (**A**) oMWCNTs, (**B**) NDs, and (**C**) oMWCNTs + NDs.

**Figure 9:**
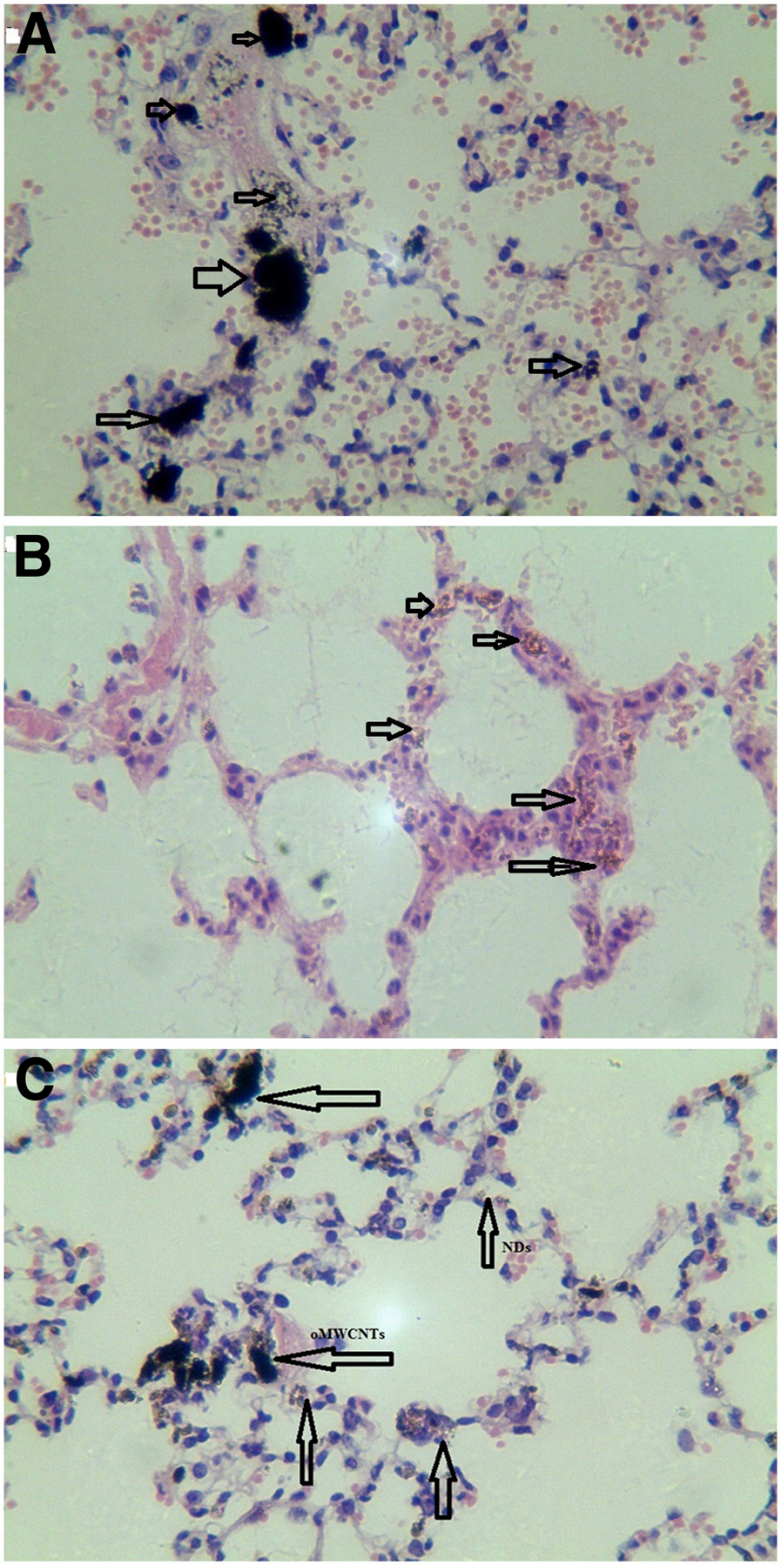
**The histology of lung tissues (×20).** (**A**) oMWCNTs, (**B**) NDs, and (**C**) oMWCNTs + NDs.

**Figure 10:**
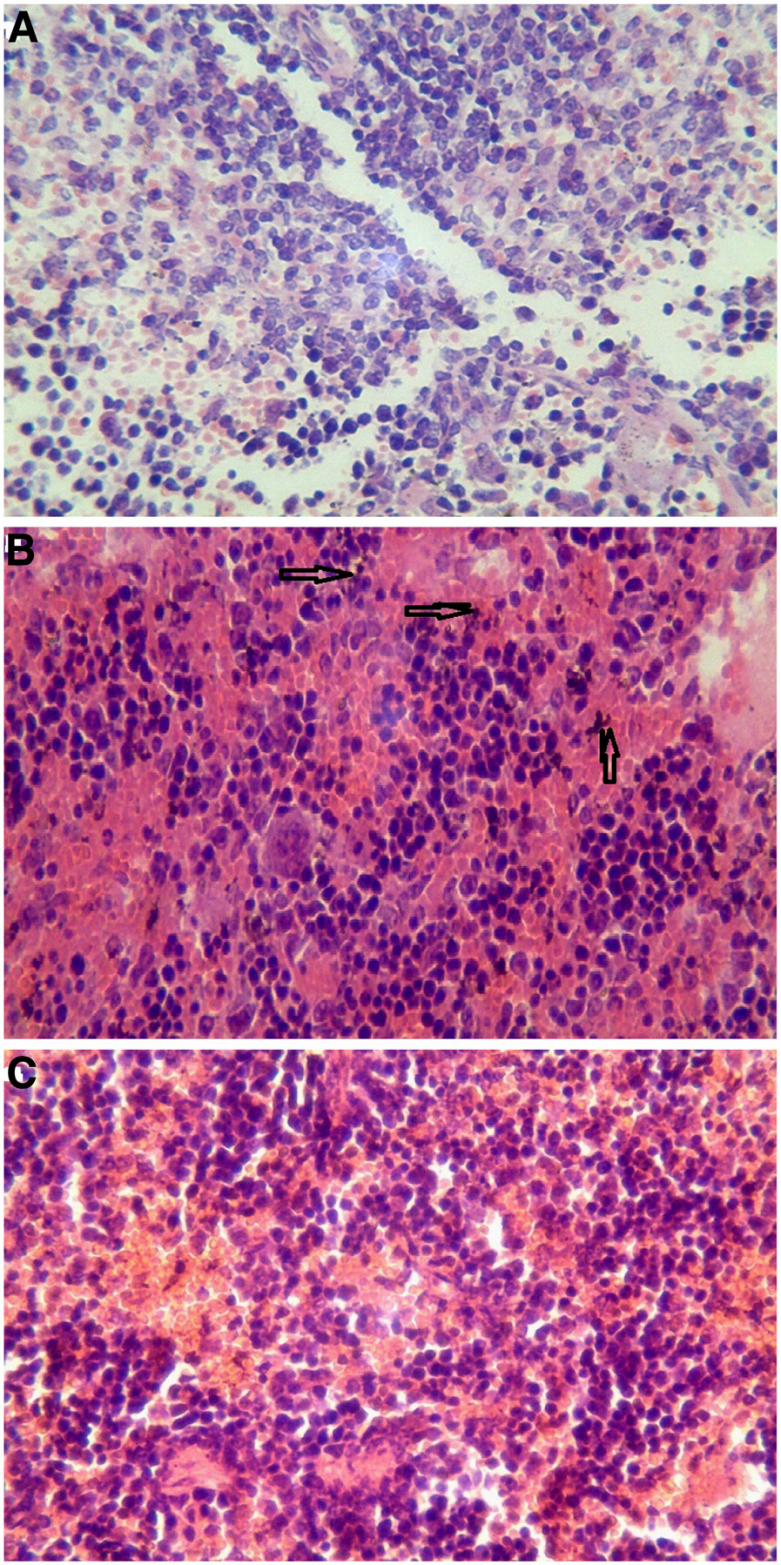
**The histology of spleen tissues (×20).** (**A**) oMWCNTs, (**B**) NDs, and (**C**) oMWCNTs + NDs.

**Figure 11:**
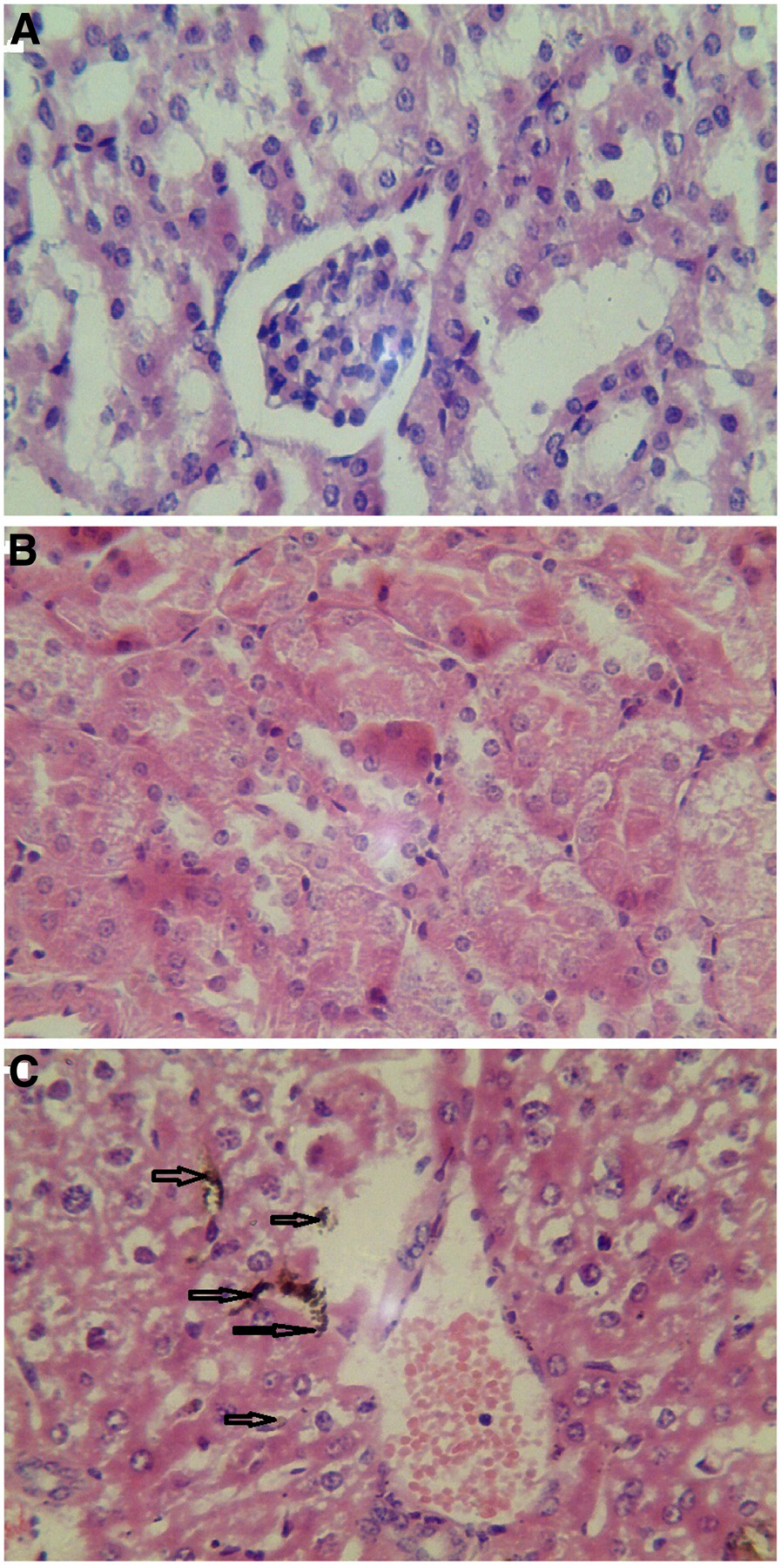
**The histology of kidney tissues (×20).** (**A**) oMWCNTs, (**B**) NDs, and (**C**) oMWCNTs + NDs.

Three groups (three mice per group) were sacrificed at 2 h post i.v. with 800 μg of oMWCNTs, NDs, oMWCNTs + NDs (pH = 7.28, *C*_NaCl_ = 0.9%, as control). Some tissues of organs, such as the lungs, liver, and spleen, were immediately collected. Those tissues were digested using 70% perchloric acid and 30% hydrogen peroxide (mixture of 1:2) [15], whereafter pH neutrality was regulated by NaOH solution and TEM (Additional file 1: Figures S3, S4, and S5) was performed. The results showed that many nanoparticles could be observed in those histological sections, which were in accordance with the results of the biodistribution exhibited in Figures 8, 9, 10, and 11. At 2 h after injection with nanoparticles, oMWCNTs in the lungs have been agglomerated into large black maculas (Figure 8A,C); NDs accumulated into brown spots in the histology, and it can be seen in the histological sections of the liver, spleen, lungs, and kidneys (Figures 8B, 9B, 10B, and 11B). However, at 2 h during co-exposure, an interesting result showed that the NDs and oMWCNTs were distributed in black macula and brown spot regions of the lung histology, respectively, and brown spots could be also observed in the liver and kidneys, but not in the spleen (Figures 8C, 9C, 10C, and 11C).

The TEM of tissues of high biodistribution (lungs, liver, and spleen) were shown in Additional file 1: Figures S3, S4, and S5. The results showed that many nanoparticles could be observed in related tissues of high distribution, and the amount of nanoparticles in the lungs was more than that in the spleen and liver post i.v. with NDs. Meanwhile, oMWCNTs and a mixture of oMWCNTs and NDs could be mostly observed in the lungs post i.v.

#### Blood clearance rate and excretion

**Figure 12:**
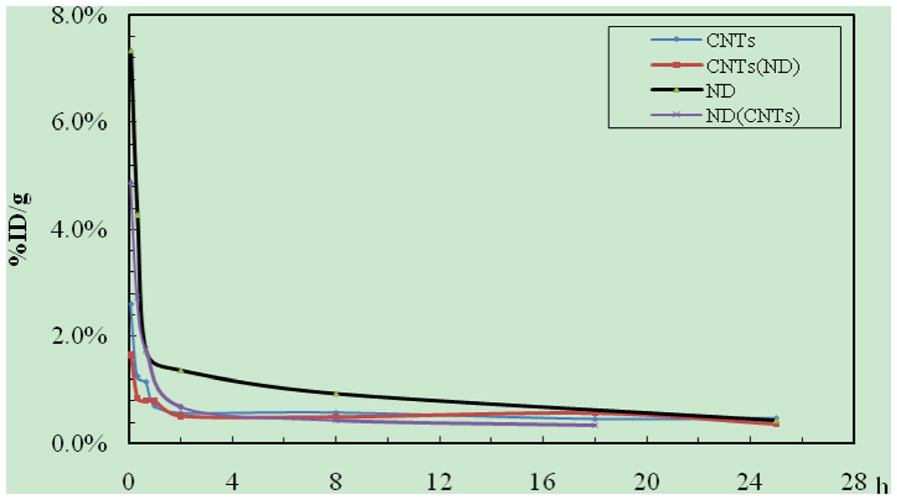
**The blood clearance rates from 0 to 28 h.** CNTs/^99m^Tc-oMWCNTs; CNTs (NDs)/^99m^Tc-oMWCNTs + NDs; NDs/^99m^Tc-NDs; NDs (CNTs)/^99m^Tc-NDs + oMWCNTs.

The blood clearance rate of nanoparticles was shown in Figure 12, and the results indicated that nanoparticles were eliminated almost completely from the blood after 2 h. The presence of NDs could enhance the elimination rate of oMWCNTs from the blood, but the excretion trends of combined injection were similar to that of the single one. As seen in Table 1, the presence of NDs could decrease the excretion values of oMWCNTs via urine. Meanwhile, the nanoparticles were excreted mainly by urine and some from feces (Table 2) during 25 h post i.v.

**Table 1:** Excretion of oMWCNTs/NDs with urine at various times

	**Time (h)**		
	**0 to 2 h**	**2 to 10 h**	**10 to 25 h**
oMWCNTs	7.06 ± 0.042%	0.805 ± 0.082%	1.841 ± 0.920%
oMWCNTs (NDs)	2.02 ± 0.651%	1.008 ± 0.385%	1.392 ± 0.099%
NDs	5.717 ± 2.413%	4.325 ± 1.59%	1.435 ± 0.850%
NDs (oMWCNTs)	1.075 ± 0.30%	3.557 ± 1.793%	6.366 ± 2.097%

At 0 to 2, 2 to 10, and 10 to 25 h (*n* = 6).

**Table 2:** **Excretion of oMWCNTs/NDs with feces at 0 to 25 h (*n* = 6)**

**oMWCNTs**	**oMWCNTs (NDs)**	**NDs**	**NDs (oMWCNTs)**
1.51 ± 0.984%	1.269 ± 0.366%	1.821 ± 0.34%	2.54 ± 0.475%

## Discussion

The radiotracing technique, with the advantages of high sensitivity, credibility, and freedom from interference, is a unique approach to investigate the fate and behavior of nanomaterials *in vivo*[16-18]. The radioactive tracer compound is prepared by covalent linkage between the radionuclide and nanomaterials, and stability is the prerequisite to study the behavior of the nanomaterials *in vivo*[6,16-18]. Therefore, the experiment of paper chromatography was performed to confirm the stability *in vitro*, and biodistribution was compared with histological results to examine the stability *in vivo*. The high labeling yield and good stability of ^99m^Tc-oMWCNTs/NDs were observed *in vitro* at all experimental time (Additional file 1: Figures S1 and S2.). The highest biodistribution of oMWCNTs was found in the lung tissues post injection with ^99m^Tc-oMWCNTs or ^99m^Tc-oMWCNTs + NDs (Figures 2, 4, and 5), and higher distribution of NDs could be found in the liver and spleen (Figure 3). Fortunately, the same results were observed in the micropathological section (Figures 8, 9, 10, and 11). The large black spots of oMWCNTs were formed in the lungs, and brown spots of NDs agglomerated in the liver and spleen, which were consistent with the tissue distribution results. It confirmed that ^99m^Tc-oMWCNTs/NDs had high stability and could affect trace biodistribution of nanoparticles *in vivo*.

The carbon nanoparticles, injected intravenously into the blood, will pass through the right atrium, right ventricle, lungs, left atrium, and into the left ventricle successively. In the left ventricle, it will be pumped into the blood circulation and carried into every tissue. According to previous reports, the pulmonary surfactant proteins (e.g., SP-A and SP-B) can be bonded to COOH/OH on the surfaces of carbon nanomaterials and form the complex of oMWCNTs/NDs-SP-A/B. The complex will enhance the capture ability of the pulmonary capillary bed on carbon nanoparticles with larger sizes (>1 μm) and will facilitate the elimination of many carbon nanoparticles with smaller sizes from the lungs [19]. Therefore, in our work, most parts of oMWCNTs (1 to 10 μm) connected with SP-A/B would be captured by the pulmonary capillary bed and accumulated mostly in the lungs (Figure 2). Because of their smaller size (<1 μm, Figure 1B and Additional file 1: Figure S7), NDs would pass easily through the alveolar wall of the capillary barrier and enter into the blood circulation again [18], and then into other tissues. That is the reason for the rapidly decreased uptake of NDs in the lungs after 8 h. It was reported that the high-level accumulation of NDs in the organs depended on the rapid capture of the reticuloendothelial system (RES), and RES capture occurred via opsonization, i.e., opsonins binding to nanoparticles in the plasma via recognition by phagocytes in the RES [9]. As shown in Figures 8B and 10B, NDs were phagocytized mainly into macrophages (e.g. Kupffer cells) of the liver and spleen so as to form many phagosomes. Thereby, many NDs escaped from the lungs, then bonded to opsonins in the plasma, and entered into the liver and spleen at once. It was captured by RES in the liver and spleen with a mass of phagocytes, resulting in high uptake of the liver and spleen from 2 to 24 h (Figure 3). oMWCNTs were captured by the pulmonary capillary bed and accumulated in the lungs, but a little part of oMWCNTs could be cleared and could enter into the circulatory system with the passage of time. Some sheets underwent mononuclear phagocyte system of organs (liver, spleen, etc.) [20], resulting in the increasing uptake of oMWCNTs in these organs (Figure 2).

It is reported that two possible pathways may be considered for the clearance of nanoparticles from the lungs with time [21]: (1) the nanoparticles are secreted by the alveolar macrophage as mucus though mucociliary transport to leave the lung, and (2) the interstitial nanoparticles are transferred through the lymph nodes and finally transferred into the liver and spleen, leading to the increased content of these organs (Figures 4 and 5) By comparing the analysis of Figures 4 and 5, NDs cleared rapidly from the lungs 24 h post i.v. with NDs + oMWCNTs, with significantly increased accumulation in the liver and spleen. Other than the two above-mentioned hypotheses, we considered that the main reason was that NDs could penetrate the capillary barrier in the lungs, escape easily from the lungs, are transported into the blood circulation again, and accumulate sequentially in the liver and spleen by RES capture [18].

The biodistribution of NDs could be affected by oMWCNTs and depended strongly on the dose of MWCNTs. Meanwhile, as showed in Figures 4, 5, and 9C, NDs and oMWCNTs could enter into the lungs together and are distributed to different parts of the lungs after co-exposure (black sheets was for oMWCNTs (Figure 9A) and brown spots with aperture periphery was for NDs (Figure 9B)). These results suggested that NDs connected to oMWCNTs (Figure 1C) were captured by the pulmonary capillary bed, together with oMWCNTs. Moreover, with increasing dose of oMWCNTs, more NDs would be connected to oMWCNTs and brought into the organs, resulting in the more obvious effects of oMWCNTs on the distribution of NDs. However, because of the complex physiological interaction, when oMWCNTs carrying NDs moved into the organs, part of the NDs would separate from the oMWCNTs, relocate to the connected groove in the pneumonic end of bronchia, and form brown spots. On the contrary, oMWCNTs still would be phagocytized by lung macrophages and form large black sheets [18,20]. This might explain the different sizes and nanostructures of NDs and oMWCNTs (Figures 1A,B). Nevertheless, further researches are still necessary to study the detailed mechanism.

As described in Figure 12, the blood uptake was below 1% at 1 h post injection, and its elimination trend was similar to the results in previous reports [20]: the blood uptake reached to a high value at 5 min after exposure, decreased to below 1%ID/g at after h, and then kept stable in a prolonged time. This result could be explained by the following processes. Firstly, the pulmonary surfactant proteins (e.g. SP-A and SP-B) would bind to carbon nanomaterials in the lungs. The complexes (NDs-SP-A/B) would facilitate the elimination of NDs from the lungs (less than 220 nm) and facilitate access into the blood again. However, the complexes (oMWCNTs-SP-A/B) could improve captures of oMWCNTs (1 to 10 μm, Figure 1) on the pulmonary capillary bed, leading to the primary accumulation of oMWCNTs in the lungs (Figure 9A) and eventually forming granuloma and longtime retention [19]. Therefore, the blood concentration of oMWCNTs/NDs decreased rapidly from 0 to 1 h, but the concentration of NDs was still higher than that of oMWCNTs after 1 h (Figure 12). After co-injection, a larger number of NDs were bonded to oMWCNTs, and then phagocytized or encapsulated with oMWCNTs by various macrophages in the lungs. As a result, the blood concentration of NDs was lower in co-injection than in single injection from 1 to 18 h (Figure 12). However, the nanoparticles released slowly and continuously from the stock in the tissues [6], so the blood concentration of nanoparticles was stable at a low level after 2 h post i.v. with NDs/oMWCNTs or NDs + oMWCNTs (Figure 12). Recently, Muller et al. reported that when nanoparticles reach the lungs, they are not rapidly eliminated but remain for over a period of at least 2 months [7]. Consequently, the oMWCNTs, aggregated easily with their big size (1 to 10 μm), would be retained mostly in the lungs (Figure 9A), resulting in the low excretion observed in the period of experiment time. However, the NDs with smaller sizes (approximately 220 nm; Figure 1A, Additional file 1: Figure S7) could penetrate easily through the biological barrier [18], reenter into the circulation system, and then arrive at the kidneys. Moreover, a small portion of NDs (approximately 11%, Table 1) was small enough to permeate through the glomerular pores, which have a minimum fenestra diameter of 30 nm, glomerular basement membrane thickness of 200 to 400 nm, and epithelial podocyte filtration slit width of 40 nm. However, after co-injection, NDs would bond to the surface of oMWCNTs and are held together by the lungs (Figure 4). The binding of pulmonary surfactant proteins (SP-A or SP-B) to NDs could promote their clearance rate from the lungs [19]. NDs would continuously penetrate the pulmonary capillary barrier, enter into the circulation system, and then be cleared or absorbed by other tissues. This process would postpone the time of excretion but did not change the quantity of excretion. Table 2 indicated that only a small amount of oMWCNTs/NDs was excreted with feces (below 3%), suggesting that oMWCNTs/NDs could be secreted into the digestive system with the bile through the liver and later eliminated with feces. This elimination process was extremely slow [21]. The metabolic pathways of the present study were similar as reported [16,22], mainly through the kidneys and bile duct. However, the authors only studied the interaction of NDs and oMWCNTs within a 24-h time frame due to the relatively short half-life of ^99m^Tc. Further studies on both toxicity and excretion of the combined influences during extended time phase were necessary.

## Conclusions

oMWCNTs were distributed largely in the lungs, but almost all NDs were accumulated in the liver, spleen, and lungs in single injection. oMWCNTs and NDs could be quickly cleared from blood post single or combined injection. oMWCNTs, relatively less excreted via the urine, could delay the excretion of NDs. The biodistribution of NDs depended strongly on oMWCNTs. NDs would accumulate mainly in the lungs post co-injection, which would be enhanced with the increasing dose of oMWCNTs. In contrast, NDs could not affect the biodistribution of oMWCNTs. oMWCNTs and NDs could be distributed in different parts of the tissues in co-injection.

## Competing interests

The authors declare that they have no competing interests.

## Authors’ contributions

QW and BJJ carried out the animal experiment. WJ and STL participated in the measurement of the properties of oMWCNTs and NDs. QW, LZ, WJJ, GY and WWS contributed in the drafting and revision of the manuscript. All authors read and approved the final manuscript.

## Supplementary Material

Additional file 1 **Supplementary information.** Biodistribution of co-exposure to multi-walled carbon nanotubes and nanodiamonds in mice. Click here for file
