# Supplementary material for: Biodistribution of co-exposure to multi-walled carbon nanotubes and nanodiamonds in mice
Source: Nanoscale Res Lett. 2012 Aug 23;7(1):473. doi: 10.1186/1556-276X-7-473 (PMC3478214; doi:10.1186/1556-276X-7-473)
Supplement: Additional file 1 — Supplementary information. Biodistribution of co-exposure to multi-walled carbon nanotubes and nanodiamonds in mice. [file 1556-276X-7-473-S1.doc]

**Supplementary information**

**Biodistribution of Co-exposure to Multi-Walled Carbon Nanotubes and Nanodiamond in Mice**

QI Wei^1^, LI Zhan^2^, BI Juanjuan^1^, WANG Jing^1^, WANG Jianjun^1^,SUN Taoli^3^, GUO Yi’an^1^, ^*^WU Wangsuo^1,4^.

1. Radiochemical Laboratory, Lanzhou University, Lanzhou 730000, Gansu, China.

2. Institute of Modern Physics, Chinese Academy of Sciences, Lanzhou 730000, Gansu, China.

3. School of Pharmacy, Lanzhou University, Lanzhou 730000, Gansu, China.

4. State Key Laboratory of Applied Organic Chemistry, Lanzhou University, Lanzhou 730000, Gansu, China.

Corresponding author: WU Wangsuo,

Email: wuws@lzu.edu.cn

1. **The radiolabeling yields of oMWCNTs or NDs;**


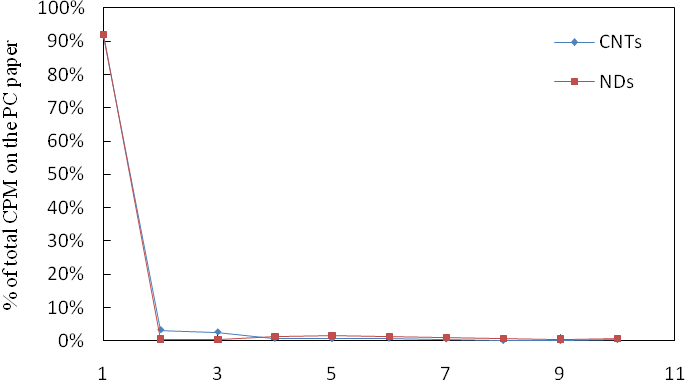


cm

Figs1. The determination radiolabeling yields of ^99m^Tc-oMWCNTs/NDs (by paper chromatography, and solvent was normal saline), it can be seen that both the radiolabeling yields were over 90%.

1. **The stability of ^99m^Tc-oMWCNTs/NDs;**


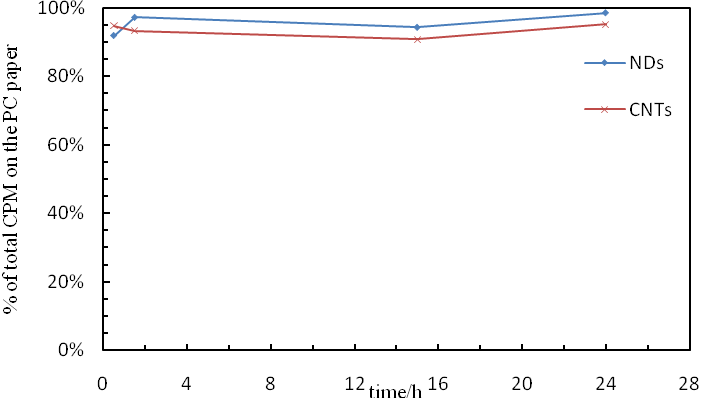


Figs2. The stability of ^99m^Tc-oMWCNTs/NDs within 24 hours *in vitro*(by paper chromatography, and solvent was normal saline), which showed all the radiolabeling compounds were very stable within 24 hours *in vitro*.

1. **The TEM of** **organization digestive juice**


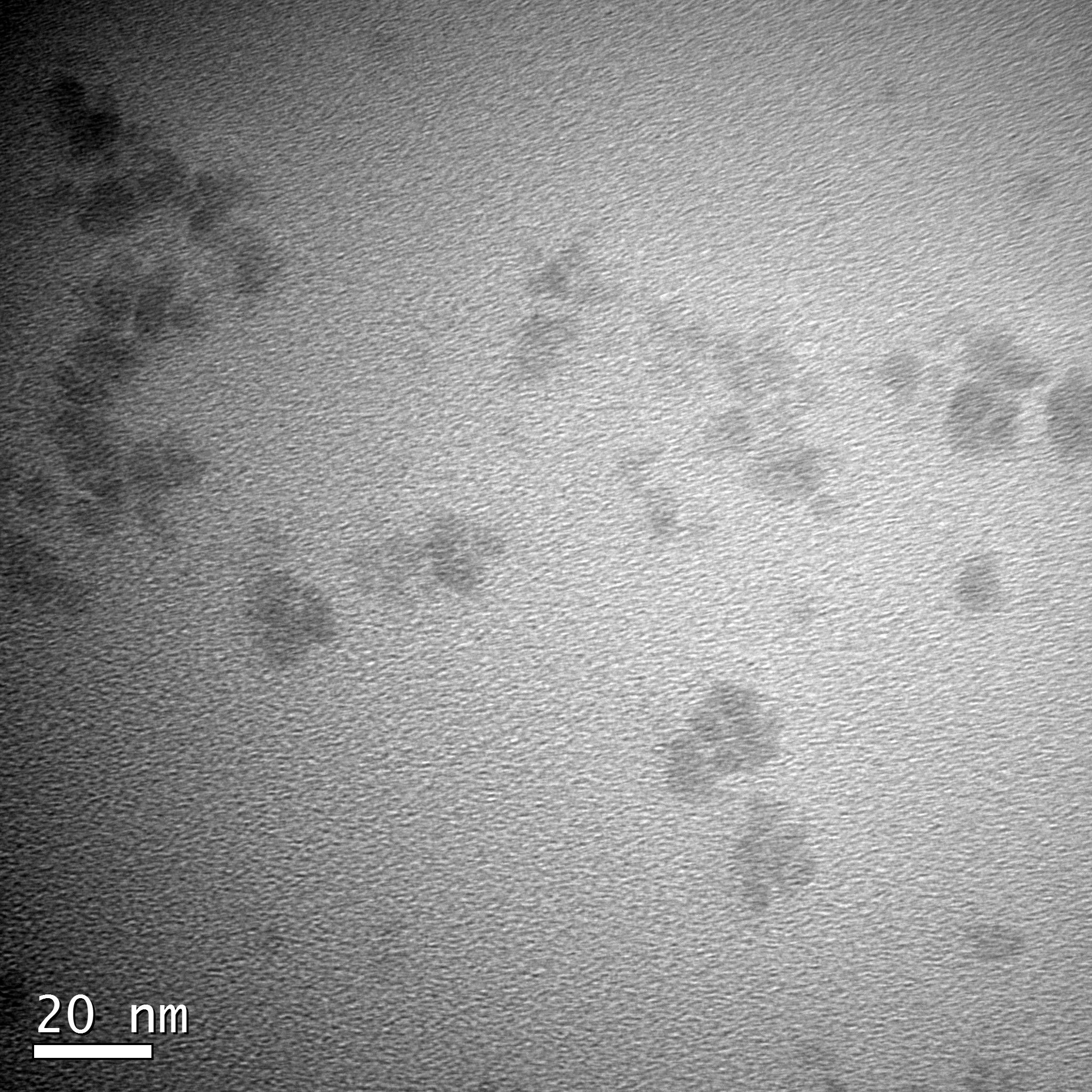


A


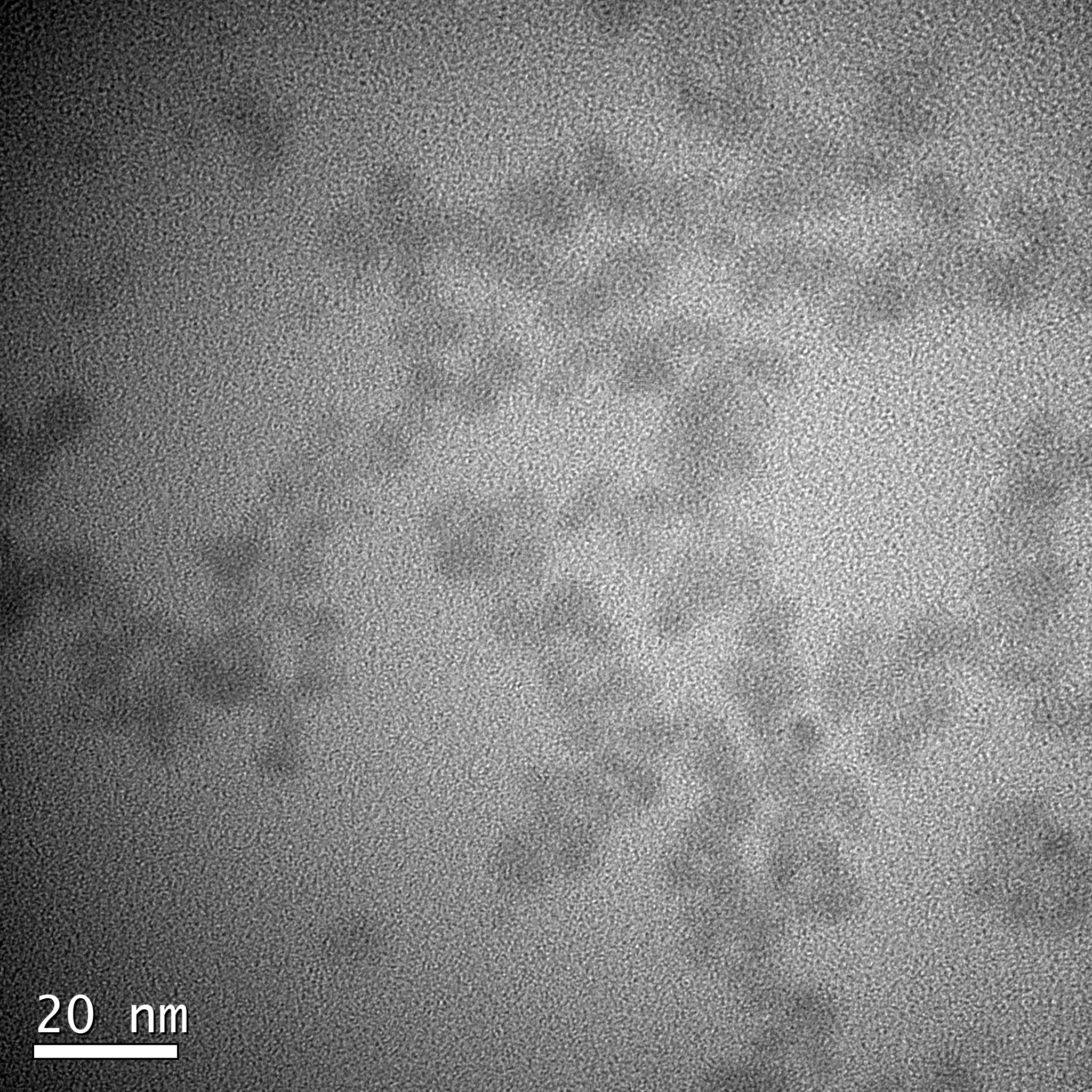


B


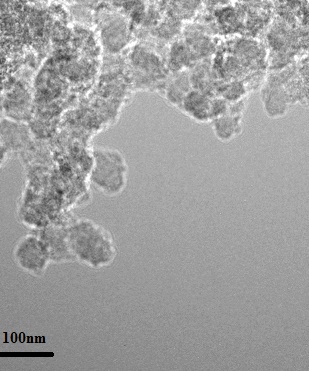


C

Figs3. The organization digestive juice at 2h after intravenous injection NDs, a for TEM of the liver, b for TEM of the spleen and c for TEM of the lung, respectively.


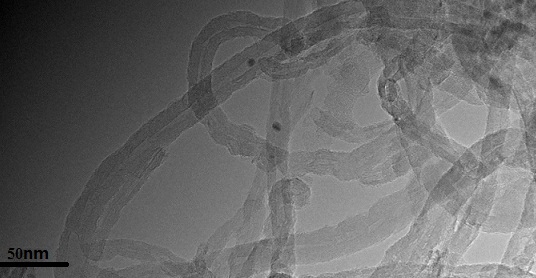


Figs4. The TEM of lung digestive juice at 2h after intravenous injection oMWCNTs


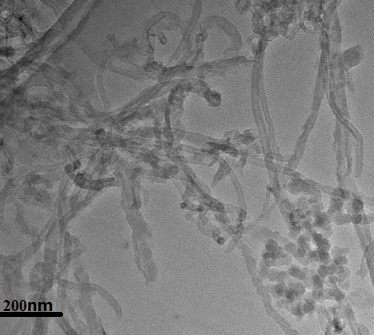


Figs5. The TEM of lung digestive juice at 2h after intravenous injection oMWCNTs+NDs

1. **The FT-IRs of oMWCNTs and NDs;**

Figs6. The FT-IRs of oMWCNTs and NDs, as to be seen from it that the absorption peak of 3400/cm was OH, and 1720-1730/cm was COOH.

1. **Particle size analysis of NDs;**

A

A

B

Figs7. Taking 2.5g/L NDs solution centrifuge (10min, 3000rpm) to collect supernatant and precipitate for particle size analysis, respectively, the result showed in A (precipitate part) and B (supernatant part), the size of supernatant was below 65 nm and precipitate was about 217nm; Then the authors calculated the mass ratio of supernatant and precipitate was 16.16%±0.8%.
